# On the Efficacy of the Sedum Acre, Linn. in Epileptic Fits

**Published:** 1805-11-01

**Authors:** 


### MEDICAL OBSERVATIONS AND CASES.

( Communicated by Dr. Arneman. )

### On the Efficacy of the Sedum Acre, Linn. in Epileptic Fits.

THE Brunonian doctrine, beatæ memoriæ, classes the epileptic fits among the asthenic diseases. However, every practitioner, who has had an opportunity of having a sufficient number of such patients under his immediate care and inspection, laments that it is out of the reach of this doctrine, either by stimulating, volatile medicines, or by the continued and progressive application of tonics, to restrain the return of this baneful disease. We therefore are obliged merely to consult the experiments and observations, made from time to time with new medicines. Two cases of Dr. Laubender, made with the sedum acre, claim the attention of the medical world.

A girl, eighteen years old, of a delicate frame, and for a number of years subject to frequent epileptic fits, was ordered to take ten grains of sedum acre mixed with sugar every morning and evening. She suffered a good deal by the medicine, being constantly sick, and she believed an emetic had been administered to her. However, she was prevailed on to continue the medicine, and five days after she took fifteen grains at once. These affected her so violently, that she complained much of her stomach and bowels, and had frequent vomitings and dejections. The dose of course was diminished, and the addition of a little cinnamon thought proper; this she bore without the least inconvenience, and continued it for some time. The epileptic leptic fit generally made its appearance every four weeks; but at the next turn, it appeared some days later, and passed over sooner. The patient gaining fresh hopes, increased the dose from one to two grains, and within four weeks she took twenty-five grains in each dose. The next fit only shewed itself, and did not come to perfection. She now took twenty-five grains, every morning and evening, and in the day an infusion of the bark in wine; at the same time a nourishing diet. The next period of the attack of the disease went over, without the least appearance. The patient thinking herself cured, wished to leave off taking medicines; this however was not permitted, and she continued till the next period, when likewise not the smallest appearance of the fit was to be perceived. The sedum acre was taken during four months, and left off in autumn. She was entirely free from epileptic fits, during a whole year; but in the next autumn she again had a fit.

A stout young countrymen, aged twenty-four years, laboured under this illness for two years, after fainting away by accident. The fit returned regularly every fortnight. Neither diet, nor change of weather had any influence upon it. The sedum acre was commenced, in doses of 15 grains each, every morning and evening; but this having very little effect, he was ordered to take, five days after, twenty grains. Constant sickness, and several times vomiting, being the consequence, he could scarcely be prevailed on to continue the medicine. The dose now was diminished, and fifteen grains with cinnamon prescribed. Two days after his body was more loose, but this soon ceased. The fits regularly appeared twice within four weeks, yet they were so far different, that the patient did not become senseless, as always had been the case before. The dose was gradually increased. During the next month he had two fits, but by no means equal in strength to the former attacks. The next month the fits did not appear at all. Having now ceased for three months, the patient was ordered to take bark with wine, and continues hitherto to be well.

